# A checklist for choosing between R packages in ecology and evolution

**DOI:** 10.1002/ece3.5970

**Published:** 2020-01-08

**Authors:** Christopher J. Lortie, Jenna Braun, Alessandro Filazzola, Florencia Miguel

**Affiliations:** ^1^ Department of Biology York University Toronto ON Canada; ^2^ The National Center for Ecological Analysis and Synthesis UCSB Santa Barbara CA USA; ^3^ Biological Sciences University of Alberta Edmonton AB Canada; ^4^ National Scientific and Technical Research Council CONICET Buenos Aires Argentina

**Keywords:** checklist, guidelines, heuristic, open source, paradox of choice, R programming language, reproducible science, statistical methods, tools

## Abstract

1. The open source and free programming language R is a phenomenal mechanism to address a multiplicity of challenges in ecology and evolution. It is also a complex ecosystem because of the diversity of solutions available to the analyst.
2. Packages for R enhance and specialize the capacity to explore both niche data/experiments and more common needs. However, the paradox of choice or how we select between many seemingly similar options can be overwhelming and lead to different potential outcomes.
3. There is extensive choice in ecology and evolution between packages for both fundamental statistics and for more specialized domain‐level analyses.
4. Here, we provide a checklist to inform these decisions based on the principles of resilience, need, and integration with scientific workflows for evidence.
5. It is important to explore choices in any analytical coding environment—not just R—for solutions to challenges in ecology and evolution, and document this process because it advances reproducible science, promotes a deeper understand of the scientific evidence, and ensures that the outcomes are correct, representative, and robust.

## Graphical abstract

Ecology and evolutionary biology comprise a rapidly changing research landscape. The programming language R is an important component of this change in teaching, research, and practice. Here, we provide a checklist to facilitate choice between similar packages in R for ecology and evolutionary biology. We also highlight the importance of increasing transparency in the practice of computation in our fields. The implication of formally contrasting packages when you make a choice to use one over another is a novel form of certainty and scientific trust.

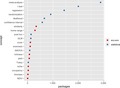

## 1 INTRODUCTION

Data are a critical form of evidence in ecology and evolution—not the only form but also an important contribution. Policy makers for the environment typically look to scientific knowledge (Rose et al., 2017), and evidence‐informed decision‐making has been frequently proposed as a mechanism to act with purpose in society to ensure that intention aligns with impact (Cooke et al., 2018). Ultimately, we inherently apply models to all the systems we examine scientifically and these can be mental, statistical, and conceptual nonexclusively (Paine, 2002). A suite of modeling techniques has been proposed for solving environmental problems that develops a framework to incorporate all stages in the process of fitting models to environmental challenges that engages with stakeholders and promotes frequent reexaminations of a model (Parrott, 2017). A methodological workflow for models is an appealing paradigm because science is iterative and reproducibility in all domains of inquiry is a critical issue (Reed, 2018). We are at a crossroads in global environmental health (Dalby, 2015; Perring, Erickson, & Brancalion, 2018) and in how we do science with the open science movement growing (Nosek et al., 2015; Tennant et al., 2016), increasing availability of data (Hampton et al., 2013), and increasing accountability of science to society (Sequeira, Bouchet, Yates, Mengersen, & Caley, 2018; Sutherland, Fleishman, Mascia, Pretty, & Rudd, 2011). Necessity is the mother of invention, and we not only have the imperative but also many of the tools we now need to do better science. Careful inspection of how we use contemporary scientific tools and methods will improve evidence production and compilation.

A fundamental element of workflows in ecology and evolution is the analysis of data. R is an open source and free programming language that is a working environment and a toolkit to do statistics in addition to many other data science and coding possibilities (R‐Core‐Team, 2019). In ecology and evolution, R has become the *lingua franca* with over 60% of 60,000 articles in these domains reporting its use by 2017 (Lai, Lortie, Muenchen, Yang, & Ma, 2019). This is a phenomenal opportunity for collaborative science, community building, and new forms of shared methodological literacy (Knuth, 1992). This is not to say that you must work in R to promote open and reproducible science. It is, however, one of many launchpad opportunities for open science because in coding our data with scripts, similar to field and lab notebooks that include annotation and description of the process of science, we can increase transparency in data and statistical decisions supporting publications (Lortie, 2017b; Lowndes et al., 2017). The code can be disseminated via version control ecosystems such as GitHub, Bitbucket, Beanstalk, or shared data and code repositories such as Zenodo, Figshare, The Knowledge Network for Biocomplexity, and Whole Tale. The contemporary culture of science expects that the code is shared and published (McNutt, 2014), and these changes in academic practices are common in ecology and evolution. Better reporting of the choices we make in analyzing data with code is also a stalwart against public criticism and uncertainty.

The R environment, without additional packages installed, is termed base R. Its default provides a set of functions for graphics, statistics, and data manipulation. However, packages offer extensions to these methods, can improve execution time, or make certain operations more accessible and sometimes simpler. Packages can also reduce coding errors and provide more efficient analyses. The R programming language has thus become a massive and distributed ecosystem comprised of the base language, packages, users, discussion and support forums, web applications, and developers. The developer component generates the packages that augment and support the functionality of R. There are over 17,000 packages listed on the R repository CRAN, and there are other relevant repositories to the natural sciences such as Bioconductor. A package in R is installed by the user and can provide additional and novel functions or tools to complete tasks such as manipulate data, visualize data, collect data, do statistics, or practically any number of conceivable functional interactions with digital assets not limited to datatables. This explosion of capacity is positive and enables rapid and hereto multifold progress. A total of 2,400 packages comprising over 3,000 functions have been used in ecology and evolution, and the journal Methods in Ecology and Evolution for instance had the highest reported proportionate usage of all journals recently examined in a recent study (Lai et al., 2019). Nonetheless, many packages does not necessarily equate to many fitting nor facile choices when functions from packages overlap. The paradox of choice has been noncausatively described as an overload or stressor to individuals when many different products fulfill the same purpose (Schwartz, 2004). We used the paradox of choice in a parochial sense here only and propose that whether few or many choices, it is important to document and report so as to enable reproducible testing by others and critical evaluation. We are not proposing that many related packages or functions are problematic but that there is a fortuitous chance to enable even greater transparency and perhaps even deeper analytics and critical thinking when choice is present (see Section 3 below). We do propose that users briefly describe how they select one package over another to complete a task in R in their published workflows or methods, and to facilitate this process, we provide a chooser checklist inspired by this abundance. Data and decision sciences are different domains but intersect in this specific context. Data science works with the data as evidence and certainly includes decision‐making (Grolemund & Wickham, 2016a) while decision sciences examine the process of making decisions to provide insights and formalize the process (Tversky & Kahneman, 1974). Decision sciences are a rich field that enable consequence analyses of decisions (Fang, Hsu, & Lin, 2019) and contrasts processes of making decisions “naturally” as individuals relative to organizational decision‐making (Gore, Banks, Millward, & Kyriakidou, 2006). This field can also support better programming through formal algorithmic contrasts of multi‐criteria decision analyses (Colapinto, Jayaraman, & Marsiglio, 2017). This knowledge commentary is not, however, a prescriptive list or step‐by‐step recipe/workflow that will guarantee selection of the most correct package. There are likely many paths to correct (and varied) solutions in ecology and evolution with data. We do propose that these ideas guide a necessary reflection prior to choosing between two or more packages that perform seemingly similar analyses in R.

## 2 CHECKLIST

**Figure 1:**
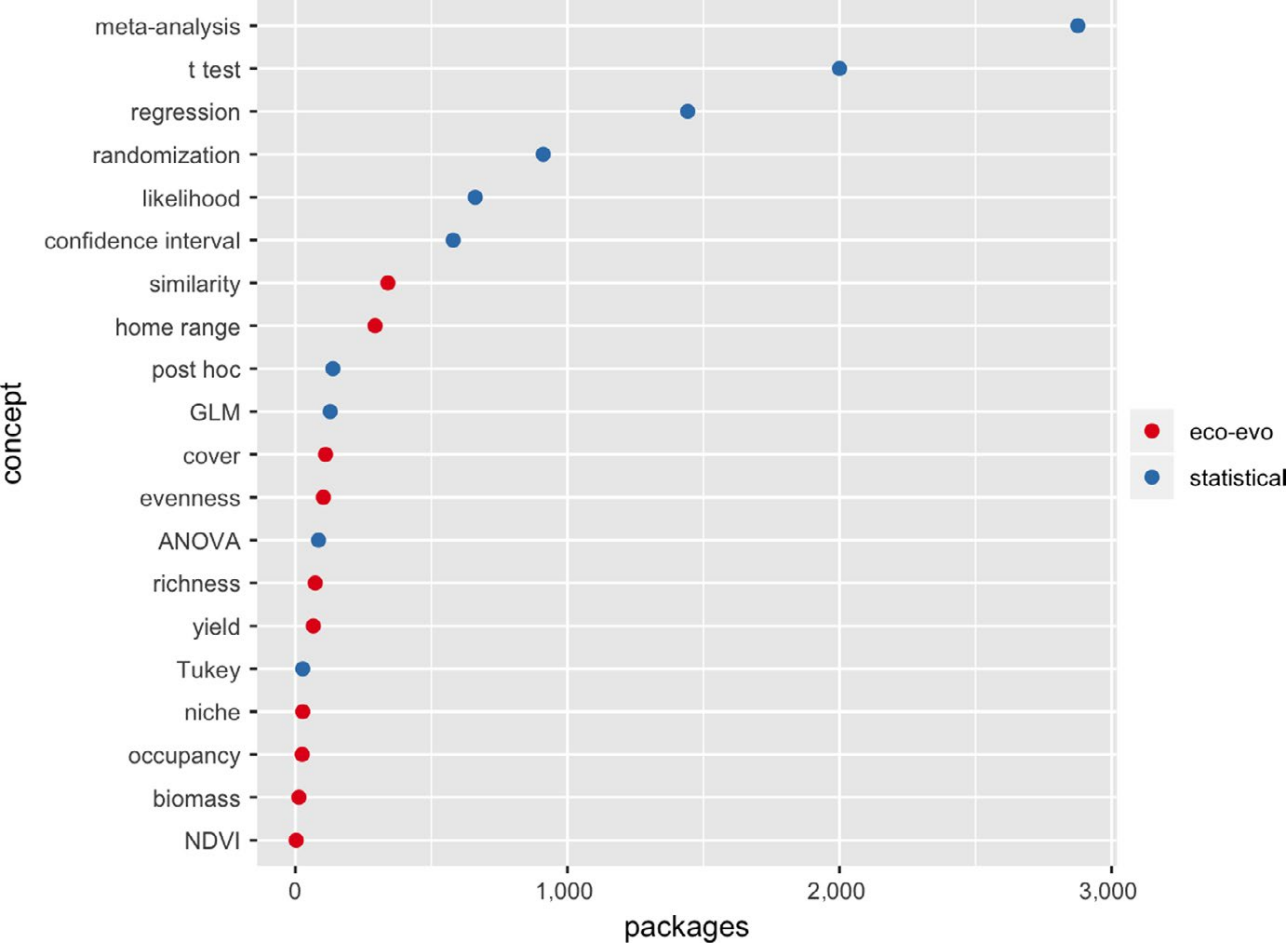
The number of R packages for common statistical and ecological/evolutionary concepts. The estimates for each term were generated using the R package “packagefinder” to search CRAN directly from the R console. The minimum number of packages returned was 3, and the maximum was 2,876 effective July 2019

When opportunity knocks, embrace the “choose your own adventure” of different R package offerings. Many of the common model‐fitting approaches in ecology and evolution have numerous packages that provide related functions. A search on the Metacran engine for common analyses such as post hoc, regression, and ANOVA returns 138, 1,442, and 85 packages, respectively. This site scrapes the entire CRAN repository, and these checks were done July 2019. The number of packages increases rapidly. More formally and reproducibly, there is an R package entitled “packagefinder” that accesses CRAN directly from within the R console to search for packages by key terms or author names (Zuckarelli, 2019). Using this mechanism, we searched for common statistical terms and representative ecological and evolutionary concepts to explore potential redundancy. At least three packages were returned per instance but typically many, many more with up to 2,876 packages listed for a single concept (Figures 1 and S1). This does not necessarily indicate that each package will provide the desired function but that the documentation mentions this term (similar to bibliometric searches of peer‐reviewed publications wherein mentions vs. relevancy are not always directly related). Typically, however, more specific eco–evo terms were associated with lower numbers of packages (GLM with a poisson fit and log‐link function, Chi‐square = 2.13, *p* = .0001, and post hoc contrast for means at *p* < .05). However, additional searches for concepts such as phylogenetics, diversity, or network analyses frequently suggest that there can be over 100 packages to review or check for the desired function or specific analytical need. A global, more generalized search for “ecology and evolution” using “packagefinder” conservatively returned a net total of 91 packages (data: Lortie, 2019a), and the top ten most downloaded packages estimated using another R package that retrieves download statistics (Yu, 2017) from within this list comprised a total of 989 distinct functions alone (data: Lortie, 2019b, code: Lortie, 2019c). Choice certainly applies in the analysis of ecology and evolution evidence. The capacity to critically contrast opportunities is thus highly relevant, and a very fitting example is meta‐analyses because they are increasingly common in the natural sciences (Cadotte, Mehrkens, & Menge, 2012; Lortie, 2014). A recent review contrasting the relative strengths of R packages compared a total of 63 different packages that directly provide functions for meta‐analytical statistics highlighting the need for thoughtful, structured comparisons (Polanin, Hennessy, & Tanner‐Smith, 2016). Hence, it is not unreasonable to assume that many of the digital‐evidence challenges we face can be resolved through a package, that is, a set of functions tested and compiled, and that there are likely to be at least several options. Furthermore, inspection of the 100 most frequent terms used to describe the functions provided by the top 10 ecology and evolution packages downloaded from CRAN to date strongly suggests noticeable overlap at this more resolved level of functional computation (Appendix S2). The ROpenSci nonprofit facilitates R package reviews submitted and registered within their community to assess and inform efficacy of some of these choices (rOpenSCi, 2019). Numerous forums including Crantastic support less structured reviews and discussion more broadly. Finally, in a text describing best practices for efficient coding in R, several criteria were also proposed for selecting packages including maturity of package, active development, extent of documentation, and frequency of use (Gillespie & Lovelace, 2017). These considerations support the development of a more extended set of ideas to contemplate, and here, we provide a snapshot of the attributes many natural scientists now appraise in this step of analyses using R (Table 1). Not all are critical in every context but most warrant some level of introspection. Ultimately, the most correct and relevant output for your challenge should be the key criterion. Just because you can do it in R or any other ecosystem does not mean you should. This suggests that the first step in the reflection and review process focuses on a check for the validity of your general decision to handle the data and evidence in a specific workflow and additional research to ascertain the most appropriate associated outcomes and estimates of accuracy. Apply decision sciences to your challenge before data sciences (Moallemi et al., 2020). Then, consider (re)iterative loops in this reflection process between the data and your decisions.

**Table 1:** A set of criteria to consider in contrasting packages in the R open source programming language and environment

Item	Criteria	Necessity
1	Maturity	Yes
2	Active development	Yes
3	Recently updated	Yes
4	Documentation available	Yes
5	Used/published in similar projects	Yes
6	License	Yes
7	Semantics intuitive	Yes
8	Functions that get the job done	Yes
9	Arguments to support your needs	Yes
10	Dependencies reported/reasonable	Yes
11	Package maintainer on GitHub	No
12	Vignettes available	No
13	Aligned workflow	No
14	Contemporary grammar	No
15	Visualization options	No
16	Connects to other packages	No
17	Speed	No
18	Source code	No

Note

A package can augment and support almost all steps in a scientific workflow, and the wealth of packages developed are extensive and frequently overlapping in the functions provided. To facilitate open and impactful science, a total of 18 criteria are proposed as a mechanism to contrast two or more packages that share similar/related functions. Necessity is proposed to weight the relative importance of each criterion.

A total of 10 criteria are proposed as necessary reflection points when reviewing two or more packages that most likely perform similar functions. Briefly, the first four items leverage the ideas previously proposed in efficient coding practices for R users (Gillespie & Lovelace, 2017). The goal is to extend and link these items to a larger set of outcomes in addition to efficiency. Maturity is proposed as a criterion. It is defined as both time since first release and the extent that a package has matured through use, development, and subsequent releases. A package that has a reasonable frequency of releases suggests that the package is maintained for stability and code bug fixes (date and version releases are listed on CRAN for every package). CRAN also drops older packages if they fail with newer builds of base R. More simply, this general criterion describes duration since the first stable public release to CRAN. Dev versions for many packages can be installed from GitHub but do not come with the guarantee of longevity for that package as a whole or for a specific function. The second item, active development, describes the last date of release and is an important consideration because the programming language R changes and is routinely updated. Statistics can evolve too (Hector, 2017; Sequeira et al., 2018), and relatively older packages can become a challenge to implement as time progresses for many reasons. Finally, “recently updated” is also a valid check for a package. Maturity is thus the first date of stable release to CRAN, active development is the last date of release, and recently updated is the relative difference between last release date and the current date that you are considering use of a specific package. These items collectively suggest that the release history of a package can illuminate its resilience and stability. Newly developed packages can do new and important things too, and the intent is not to bias against these offerings. The action proposed here is to examine history even for relatively recent offerings to ensure that stability and some sense of reasonable longevity are provided. Reanalyses at a later date or with new data, the process of peer review if the work is included in a potential journal publication, and subsequent introspection of ideas in ecology and evolution all suggest that at least limited certainty is a prudent strategy. A secondary action can be a contrast of outputs from relatively newer versus more established packages to explore differences. Then, check the science.

We propose that other meta‐data attributes of packages are also important criteria in addition to release history. Documentation in the R ecosystem is enabled through manuals and vignettes published on CRAN. A check for the presence, quality, and clarity of these resources can inform the potential user experience. Furthermore, publications describing the use of packages in ecology and evolution strongly suggest that the package will be functional and potentially appropriate to your own adventure, that is, see the R package codyn on CRAN for community dynamic measures with documentation and a peer‐reviewed publication to describe it in depth (Hallett et al., 2016). There are many other examples also published in the journal Methods in Ecology and Evolution (Duthie et al., 2018; Harmer & Thomas, 2019; Remelgado, Wegmann, & Safi, 2019; Wubs et al., 2019). Another important attribute of packages is the frequency of use. This can be assessed through download statistics, analyses of popularity reported online or reported in synthesis publications (Lai et al., 2019), or through inspection of the description of methods in related primary scientific studies. These checks do not necessarily mean that a package is suitable for your specific task but wide adoption can indicate that a package functions well for its express purpose. Finally, a meta‐data property of packages that warrants inspection is the license. The license grants rights of use, and typically packages in R are associated with either GPL‐2 or 3 (general public licenses). The more recent iteration of this common open source software license includes an explicit patent license that provides additional protection and freedom to the (re)users. A brief review of licensing is suggested particularly for developers, and an increasing component of early career researchers in ecology and evolution participate in R development. Considering the attributes associated with a package that do not directly relate to the functions needed can seem like an inefficiency but do highlight potential frictions in use, reuse, or innovation at later stages in your workflow. The capacity to replicate analyses and interpret epistemological validity can also be a critical socioscientific component to decision‐making (Fang et al., 2019). A relatively more popular, documented, or openly licensed package is not necessarily the most accurate, but shared wisdom within a field of study and community of scientists is an important pathway to consolidation and to discovery too. These attributes signal some measure of that capacity, and a key action to consider is a critical comparison between the package description and context of use with developments in the theory of ecology and evolution. We build on the advancements of others individually and through collective efforts in science (Bornmann, Moya Anegón, & Leydesdorff, 2010) and in coding.

The final four necessary criteria proposed describe the relative likelihood for a specific R package among a set to get the job done. Statistical semantics is a formal term (Rekabsaz, Bierig, Ionescu, Hanbury, & Lupu, 2015), but here, we use the concept of semantics more broadly to describe the meaning of the terms employed to label functions and statistical tasks (Soyer, 2017). A function is an action or process in R, and the language used in the package to describe functions is more likely to support the task at hand if the terms match those that refer to the biology or ecology of the system in study, that is, diversity, richness, abundance, and habitat occupancy. The same applies to the objects in R manipulated by a package and the assets or evidence for a project that one seeks to examine. Ideally, the documentation should not be a struggle to comprehend, and terms that describe statistical functions and objects align with the wider analytical and scientific literature. Time series analyses in R are a great example of excellent alignment between code and knowledge (Killick, 2017; Lortie, 2018). Some packages clearly cite technical and methodological publications including texts, and the provenance of the conceptual development is clearly delineated. Importantly, the functions and arguments provided by a specific package must support your needs and evidence. Different packages can have functions with relatively more arguments, that is, specifications that can or must be supplied, and different requirements for the form and structure of your evidence such as the data formats and organization. Typically, this can resolved with wrangling and tidying (Grolemund & Wickham, 2016b; Lortie, 2017a), but this is not guaranteed. The benefit of some packages is that they can handle assets and data that are unique. Dependencies between data and code from a package also extend to dependencies between packages in R. Packages can source/reuse functions from other packages, and this is not necessarily problematic but should be inspected. Furthermore, connection to other related packages (proposed as a preferred but not critical criteria later in Table 1) can be advantageous. Undue dependencies are a relative concept for the experience of the user, and parsimony is preferred. Each project will nonetheless vary in the extent that this resilience is influenced by the anticipated capacity for maintenance of supporting packages. For example, the tidyverse is a set of packages for reading and manipulating data with supporting visualization functions that is well maintained. Increasing related development also includes many other packages labeled “tidy” that assume a consistent data structure and flow of analysis termed grammar (and also user familiarity) (Wickham, 2016). This grammar initially described only the graphical thinking of layering visual elements on top of another and building up plots, but it has trickled to many other functional roles for contemporary R package development and is thus proposed as another preferred criterion. Alignment between packages will facilitate a more integrated project‐level workflow (and less data reformatting) and suggests that a priori consideration of the analytical scaffolding needed to build out the R component of a scientific project is time well spent. That said, the goal should be to embrace pluralistic pathways to discovery in science and in data science choices. Alignment should not become constraints. At least three action items are evident from these criteria. Firstly, ensure terminology and meaning of your scientific concepts aligns with the functions described in an R package. Secondly, choose packages with grammar and functional steps that best support your thinking and evidence. Finally, consider inspecting the package dependency of these supporting packages too. Build choice on a solid foundation conceptually and programmatically through integrated thinking and coding.

There are several other remaining proposed criteria to review if one is in the fortunate position of having numerous viable packages that satisfy the resilience, need, and integration criteria sets proposed above. Familiarity with the package developer(s) is not critical but can be useful. Domain‐level expertise such as a wildlife ecologist developing packages for the field of study and relevant data is a telltale sign of a good fit for your wildlife project. There are many examples such as an R package developed for a resource selection function model to examine habitat choice in animal populations (Solymos, 2019), and the approach was discussed in peer‐reviewed publications prior to the package by the developer (Sólymos & Lele, 2016). Recognized expert in statistics or data science and making contributions to the field of discourse in some capacity in addition to R packages is a further positive indicator. Discussion can be online though forums, mailing lists, and issues on repositories. In many instances, one can directly contact package maintainers. Development on a code repository can provide insights into the underlying assumptions and work by the maintainers. Vignettes in R are also a similar criterion to consider because they typically provide a worked‐through solution for that package with a representative dataset (often included with the package). This can be informative to learners that prefer this approach to problem‐solving in addition to reading a manual. Learn‐by‐doing is a common learning style in computational biology. In addition to package review or inspection, the key action would be to test them with your code or run the code provided in the respective vignettes. Visualization can be achieved by generic packages such as ggplot2 that handle an incredible breadth of data types (Wickham, 2016), but specific plots from packages can provide a rapid insight into a particular or more idiosyncratic phenomena. Plots for exploration, interactivity, communication with stakeholders, or publication can potentially require different packages. Next, speed to execute a process in R can at times be a constraint and is a valid consideration (Gillespie & Lovelace, 2017). Imagery and spatial data analyses are rapidly improving in R, and there have been significant revisions to packages such as raster and rgdal to become more efficient and more effective in memory allocation (Lovelace, Nowosad, & Muenchow, 2019). The landscape of packages associated with mapping in R is also increasingly diverse and varied and a challenge to navigate in some contexts. Finally, a dive into the underlying code can be illuminating for at least two reasons when contrasting packages. Firstly, some packages are written exclusively using R but others use C++ or other programming languages such as Python for the source code. If you have development needs, a package that is coded strictly in R can have the advantage of being more familiar and modifiable by the user whereas C++ code can be more efficient but foreign to many native R users. Secondly, the underlying code can be important because the similar or even same function such as a generalized linear mixed model can be achieved through established mathematical approximations or through simulation and permutation (Bolker et al., 2009). Documentation may not always be entirely transparent. Sensitivity in how a package handles the data or reports and estimates an outcome can thus vary between packages. Choice is thus an actionable opportunity to vet accuracy. In summary, these additional criteria are more nuanced but have implications on your capacity to run the code, edit the process, or assign confidence to the underlying assumptions. You spent time to get the data. Now, spend the time to examine your decision quantitatively and pragmatically if you can.

## 3 IMPLICATIONS

The intent of this checklist and heuristic for those that elect to use R in ecology and evolution is not to add more work to the process but to promote a wider and deeper view of this ecosystem. One does not have to work only in R to support open science or reproducibility. Critical thinking must be a component of all endeavors in ecology and evolution (Facionie, 2017), and important choices in data sciences can engage with decision sciences by using criteria that promote reflection and structured contrasts of varied options (Berkeley & Humphreys, 1982; Neale & Northcraft, 2005). Every project is an opportunity to advance better thinking and document choice so that others can replicate findings—including you. The benefits of this reflection process can thus be direct and indirect. Exploring different related analytical solutions provides a deeper understanding of evidence and will advance statistical and scientific reasoning (Smith, 1998). These contrasts, when needed, also prepare one for the peer review and dissemination process. This is a novel form of certainty and scientific trust, and reporting these choices will help anticipate the devil's advocate including challenges to sensitivity in outcomes and models. Ideally, there is only one correct answer, and the package you select produces this outcome. However, this perspective is a bit naive, and a recent paper proposed that “all ecological models are wrong, but some are useful” (Stouffer, 2019). This is exactly when it is most critical to carefully review the different outputs from similar packages. Directly, even cursory contrasts of packages will enhance exploratory data analyses and strengthen final reporting. Connecting these package contrasts to fundamental scientific theory will consolidate linkages between concepts in data science and innovations in the field of inquiry. Indirectly, this supports and promotes engagement and discourse with the development community and can introduce a new feedback loop into our community for enhanced methodological discoveries. These contrasts will profoundly improve development, testing, and collaboration in an already thriving community of scientists and coders (Markowetz, 2017). Every tool is not a hammer in R, and fitting the right tool to the right challenge provides coherence to ideas and concepts and elevates discovery. Describing how we choose study sites, subjects, and experimental methodologies is commonplace and routine in science. In many contexts, we also explore controls. Reporting and testing different R packages (or functions written for a specific project in base R) are natural extension to the scientific process. This process benefits the community at large ‐ developers and end users alike—because we highlight and promote better and more useful package development through scientific reasoning and observation. A single modality or R package need not always prevail, that is, one ring to the rule them all. Being mindful of the fit of a specific package to your needs and challenge is a form of user experience feedback and experimentalism that can shape how you do your data work and makes the implicit more explicit in your workflows. Knowing that you have chosen wisely will more likely conclude your adventure in R packages and science with a happy ending.

## CONFLICT OF INTEREST

None declared.

## AUTHOR CONTRIBUTIONS

CJL conceived the idea and wrote the manuscript; CJL did the analyses; CJL, JB, AF, and FM reviewed the ecology and evolution R packages. All authors contributed critically to the drafts and gave final approval for publication.

### OPEN RESEARCH BADGE

This article has earned an Open Materials Badge for making publicly available the components of the research methodology needed to reproduce the reported procedure and analysis. All materials are available at https://zenodo.org/record/3333045#.XdHVEy2ZMWo.

## Data Availability

Data are available at Figshare at https://figshare.com/articles/Ecology_and_evolution_R_packages/8813276 and https://figshare.com/articles/Top_ten_R_packages_in_ecology_and_evolution/8813174. Code is available at Zenodo https://zenodo.org/record/3333045#.XdHVEy2ZMWo.
