# Supplementary material for: A checklist for choosing between R packages in ecology and evolution
Source: Ecol Evol. 2020 Jan 8;10(3):1098–105. doi: 10.1002/ece3.5970 (PMC7029065; doi:10.1002/ece3.5970)

**Supplement 2.** A word cloud of the 100 most frequent terms used to describe functions for the 10 most downloaded ecology and evolution R packages from CRAN. All code used to collect and visualize these data archived at Zenodo. See literature cited for published data of complete counts of all terms and code used to compile frequencies.


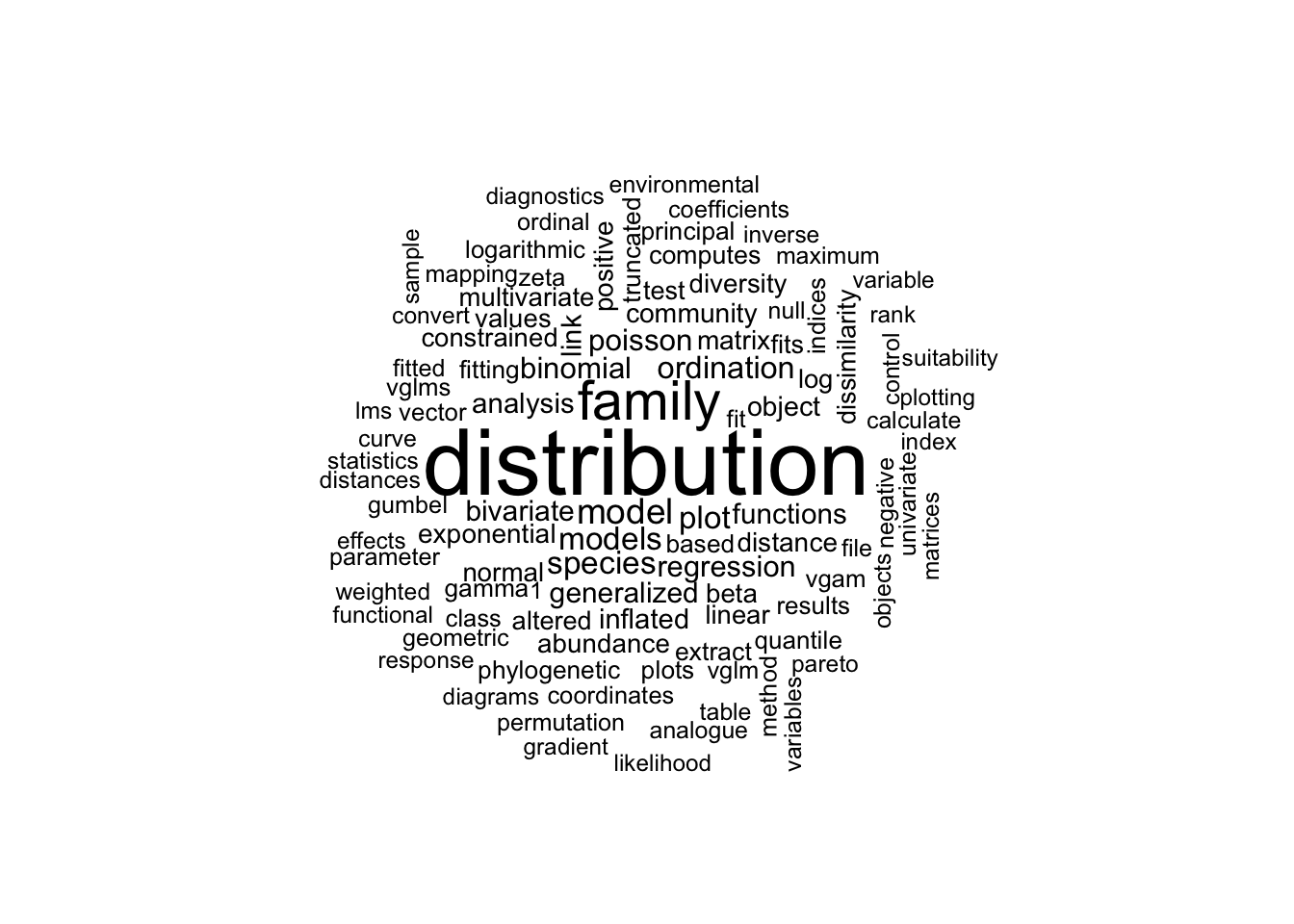

Supplement: Supplementary file 1 [file ECE3-10-1098-s001.docx]
