# Supplementary material for: A checklist for choosing between R packages in ecology and evolution
Source: Ecol Evol. 2020 Jan 8;10(3):1098–105. doi: 10.1002/ece3.5970 (PMC7029065; doi:10.1002/ece3.5970)
Supplement: Supplementary file 2 [file ECE3-10-1098-s002.docx]

**Appendix S1**. A contrast of the number of R packages associated with common statistical and ecological/evolutionary terms. Code for these searches is archived at Zenodo (see literature cited for citation and full access).

| **rep** | **specificity** | **concept** | **returns** |
| --- | --- | --- | --- |
| 1 | statistical | post hoc | 138 |
| 2 | statistical | regression | 1442 |
| 3 | statistical | ANOVA | 85 |
| 4 | statistical | meta-analysis | 2876 |
| 5 | statistical | t-test | 2000 |
| 6 | statistical | Tukey | 27 |
| 7 | statistical | randomization | 911 |
| 8 | statistical | confidence interval | 580 |
| 9 | statistical | GLM | 128 |
| 10 | statistical | likelihood | 661 |
| 11 | eco-evo | occupancy | 25 |
| 12 | eco-evo | home range | 293 |
| 13 | eco-evo | richness | 73 |
| 14 | eco-evo | evenness | 103 |
| 15 | eco-evo | similarity | 340 |
| 16 | eco-evo | biomass | 13 |
| 17 | eco-evo | yield | 66 |
| 18 | eco-evo | cover | 111 |
| 19 | eco-evo | NDVI | 3 |
| 20 | eco-evo | niche | 27 |

**Appendix S2.** A word cloud of the 100 most frequent terms used to describe functions for the 10 most downloaded ecology and evolution R packages from CRAN. All code used to collect and visualize these data archived at Zenodo. See literature cited for published data of complete counts of all terms and code used to compile frequencies.


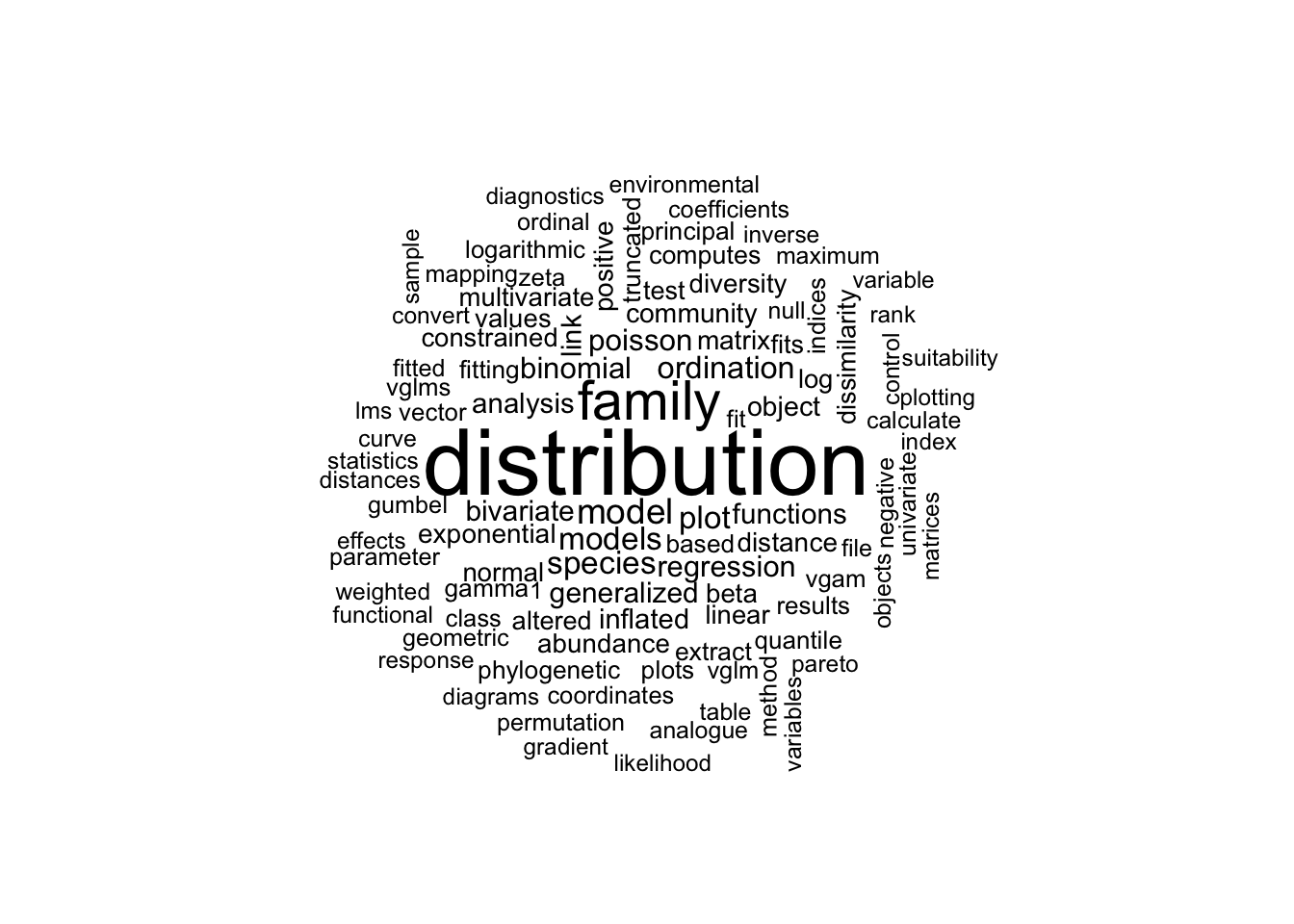


**Appendix S3.** Criteria to contrast R packages for a random effects meta-analysis and test for publication bias. There are over 100 relevant R-packages available on CRAN to conduct meta-analyses. We selected five meta-analysis packages for comparison that listed random-effect models and publication bias tests. Using the criteria listed below, we can make informed decisions on the package most applicable to our needs.

| **item** | **criterion** | **meta** | **metafor** | **rmeta** | **metaplus** | **CAMAN** |
| --- | --- | --- | --- | --- | --- | --- |
| 1 | maturity | Version 4.9-7, released 2007 | Version 2.1-0, released 2010 | Version 3.0, released 2006 | Version 0.7-11, released 2016 | Version 0.74, released 2013 |
| 2 | development | Yes | Yes | Unknown | Unknown | No |
| 3 | updated | Last version Sept 2019 | Last version May 2019 | Last version March 2018 | Last Version April 2018 | Last Version Sept 2016 |
| 4 | documentation | Yes | Yes | No, manual only | Yes | No, manual only |
| 5 | similiarity | Rest et al. (2016). Meta_analysis and meta_regression of transcriptomic responses to water stress in Arabidopsis. The Plant Journal, 85(4), 548-560. | Filazzola et al. (2019). The contribution of constructed green infrastructure to urban biodiversity: A synthesis and meta_analysis. Journal of Applied Ecology. | Unknown | Pepper et al. (2018). Telomeres as integrative markers of exposure to stress and adversity: A systematic review and meta-analysis. Royal Society open science, 5(8), 180744. | Unknown |
| 6 | license | GPL-2 | GPL-2 | GPL-2 | GPL-2 | GPL-2 |
| 7 | semantics | Yes, tutorial provided | Yes, tutorial provided | Yes | Yes, tutorial provided | Yes |
| 8 | functional fit | metabin, metabias | rma, funnel/regtest | meta.MH, funnelplot | metaplus, testOutliers | no bias test, bivariate.EM |
| 9 | arguments | Yes | Yes | Yes | Yes | No |
| 10 | dependencies | R 2.9.1, imports grid, metafor, lme4 | R 3.5.0, imports stats, utils, graphics, grDevices, nlme | R not stated, imports grid, stats, graphics | R 3.2.0, imports bbmle, metafor, boot, methods, numDeriv, MASS, graphics, stats, fastGHQuad, lme4 | R 2.1.0 |
